# Supplementary material for: The Conserved Transcriptional Activation Activity Identified in Dual-Specificity Tyrosine-(Y)-Phosphorylation-Regulated Kinase 1
Source: Biomolecules. 2023 Feb 2;13(2):283. doi: 10.3390/biom13020283 (PMC9953678; doi:10.3390/biom13020283)
Supplement: Supplementary file 1 [file biomolecules-13-00283-s001.zip › Supplementary material.pdf]

Supplementary Material

The conserved transcriptional-activation activity identified in dual-specificity tyrosine-(Y)-phosphorylation-regulated kinase 1

Xiuke Ouyang[1], Zhuqing Wang[1], BingtongWu[1], Xiuxia Yang[1], Bo Dong[1,2,3*]

Supplementary Figures and Tables

1.1 Supplementary Figures

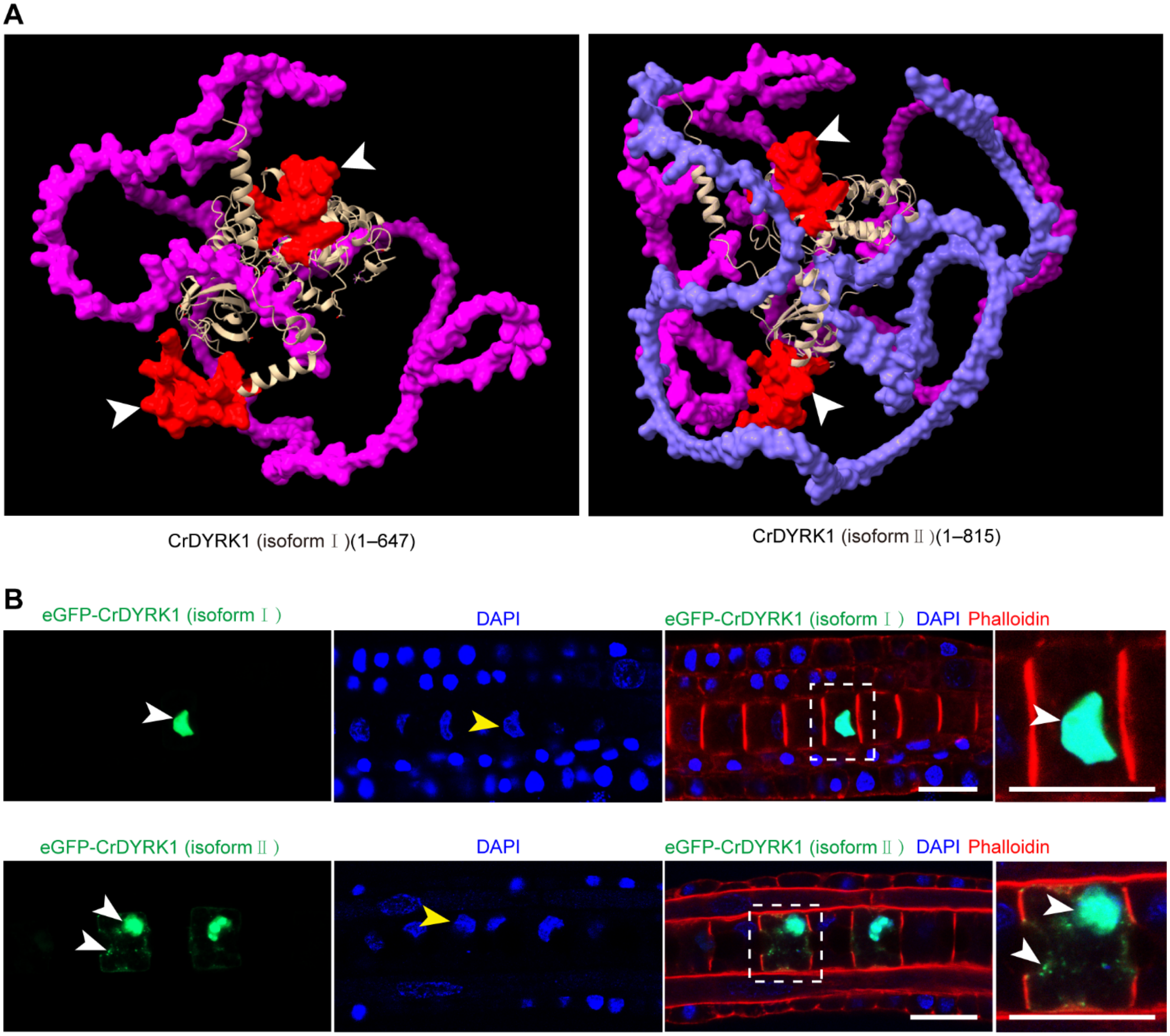

Supplementary Figure S1. The N-terminal (residues, 1–168) enhances the extranuclear localization of CrDYRK1 (isoform II). (A) The 3D structure analysis of CrDYRK1. The protein 3D structure was predicted using online tool ALPHAFOLD (https://cosmic-cryoem.org/tools/alphafold/). The N-terminal (residues, 1–168) and the C-terminal (residues, 595–815) of CrDYRK1 (isoform II) were exposed on the surface without contact physically. NLS were exposed on the surface in CrDYRK1 (isoform I) and were surrounded by residues 1–168 in CrDYRK1 (isoform II). The red represents the NLS, the purple represents the repression domain (residues, 1–168) of CrDYRK1 (isoform II), and the pink represents the activation domain. The white arrows indicate the NLS. (B) The differential localization of different CrDYRK1isoforms. Isoform I and isoform II were separately fused with eGFP and driven by notochord specific promoter *Brachyury*. The GFP signal was detected at the nucleus in the isoform I-expressing cells. Meanwhile, the GFP signal was detected at the nucleus, cytoplasm, and cell boundary in the isoform II-expressing cells. The white arrows indicate the eGFP. The yellow arrows indicate the DAPI. The white box marks the notochord cells overexpressed fusion proteins and indicated notochord cells were magnified for observing the localization. Scale bar is 20 μm.

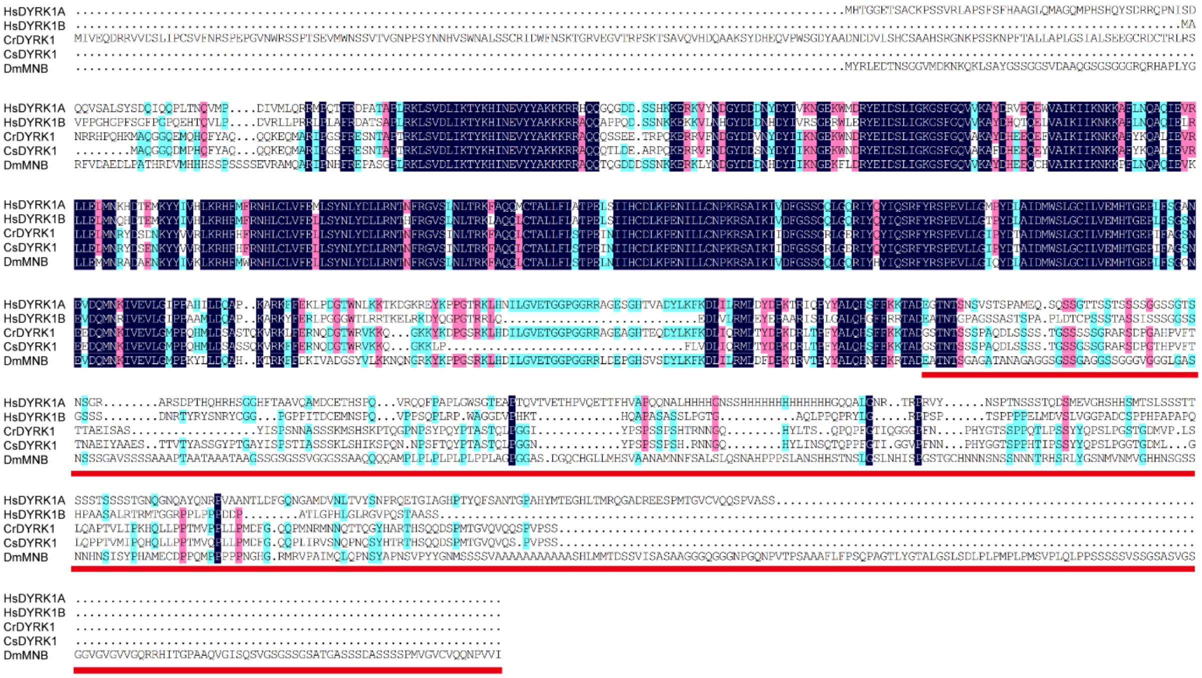

Supplementary Figure S2. The full lengths amino acids sequence alignment of DYRK1 from different species. The homologous region of DYRK1 from diverse species with CrDYRK1 (isoform I) activation domain (residues 427–647) were identified by amino acids sequence alignment. The red line indicates the homologous region.

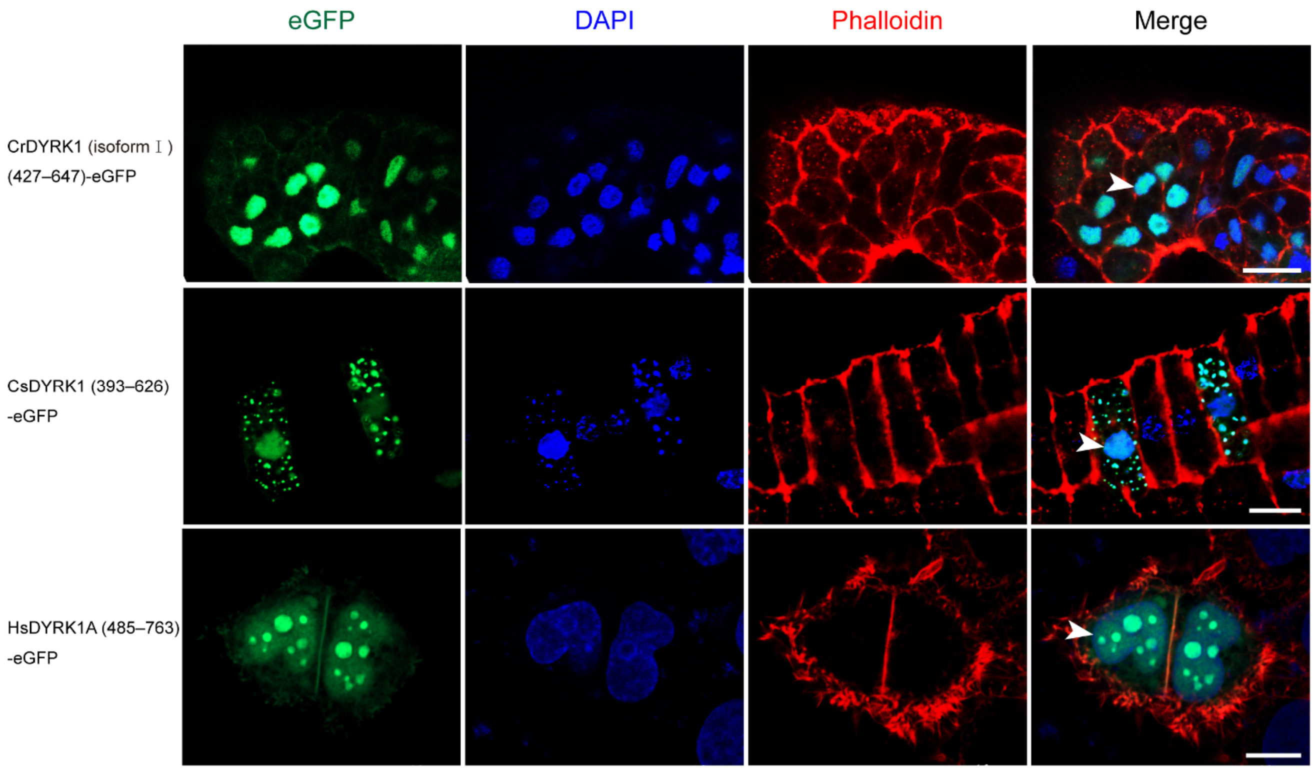

Supplementary Figure S3. The subcellular localization of activation domain of DYRK1. *CrDYRK1* (isoform I)(427–647)-eGFP was expressed in epidermis cells of *C.robusta*. *CsDYRK1* (393–626)-eGFP was expressed in epidermis cells of *C.savignyi*. *HsDYRK1A* (485–763)-eGFP was expressed in Hela cells. The result showed the activation domain of DYRK1 localized in cell nuclear in the absence of two nuclear localization signals of N-terminal. The white arrows indicate the cell nuclear. Scale bar is 10 μm.

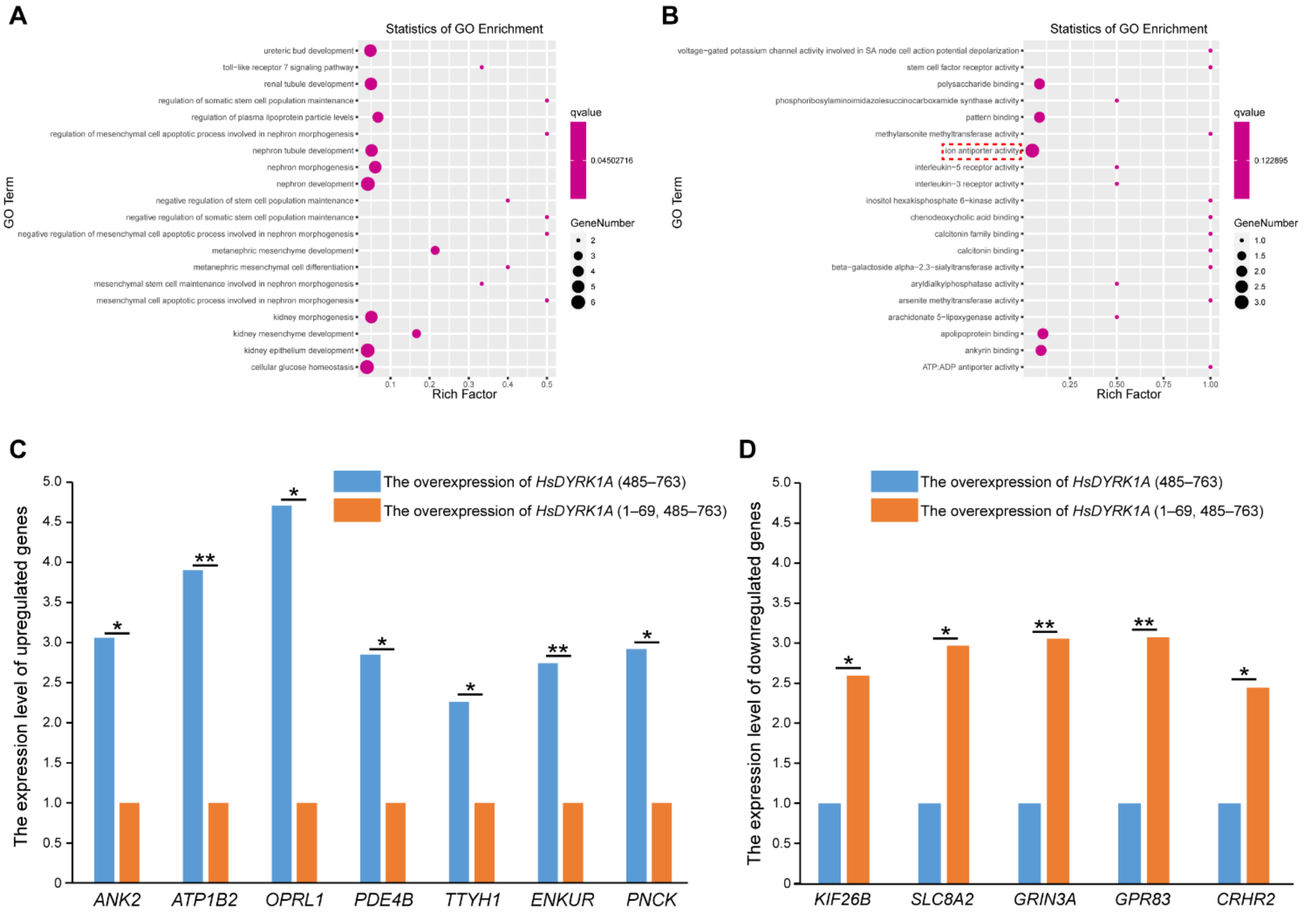

Supplementary Figure S4. The bioinformatics analysis of downregulated genes and the expression level of some DEGs. (A) The biological process (BP) analysis of downregulated genes. Downregulated genes were mainly involved in kidney development. (B) The molecular function (MF) analysis of downregulated genes. Downregulated genes mainly enriched in ion antiporter activity terms. (C, D) The expression level of DEGs. These up- or downregulated genes were mainly expressed in brain and involved in ion transport and neuroactive ligand-receptor interaction.

1.2 Supplementary Tables

Supplementary Table S1: Primers used for vector construction in this study.

Supplementary Table S2: Differential expression genes of transcriptome assay.
